# Fecal Microbiota Profiling in Indigenous Backyard and Commercial Chickens Reveals Distinct Taxonomic Signatures

**DOI:** 10.1155/vmi/3146713

**Published:** 2026-05-04

**Authors:** Abdullah Hasib, Stephen Ogada, Susan Maina, Stephen Kuria, Min-Sheng Peng, Jiangkun Yu, Sheila Cecily Ommeh

**Affiliations:** ^1^ The University of Queensland, School of Agriculture and Food Sustainability, Gatton, Queensland, Australia, health.qld.gov.au; ^2^ The University of Queensland, Queensland Alliance for Agriculture and Food Innovation, Centre for Animal Science, Gatton, Queensland, Australia, health.qld.gov.au; ^3^ Institute for Biotechnology Research, Jomo Kenyatta University of Agriculture and Technology, Nairobi, Kenya, jkuat.ac.ke; ^4^ State Key Laboratory of Genetic Evolution & Animal Models and Yunnan Key Laboratory of Molecular Biology of Domestic Animals, Kunming Institute of Zoology, Chinese Academy of Sciences, Kunming, China, cas.cn; ^5^ Sino-Africa Joint Research Center, Chinese Academy of Sciences, Nairobi, Kenya, cas.cn; ^6^ State Key Laboratory for Conservation and Utilization of Bio-Resources in Yunnan, School of Life Sciences, Yunnan University, Kunming, Yunnan, China, ynu.edu.cn

**Keywords:** 16S rRNA, chicken production system, fecal bacteria, food safety, gut microbiota

## Abstract

Farm management conditions and feeding practices in free‐range (backyard), semi‐intensive, or intensive production systems significantly influence the poultry gut microbiome, thereby impacting their productive performance and overall health. Here, fecal samples from asymptomatic indigenous backyard chickens raised in a free‐range production system, characterized by little to no biosecurity measures in place, and from commercial chickens raised in an intensive production system with enhanced biosecurity measures were subjected to 16S rRNA sequencing analysis. Taxonomic assignment identified 19 bacterial phyla, 137 families, and 238 genera. The most prevalent phyla in indigenous backyard and commercial chickens were *Firmicutes*, *Proteobacteria*, *Bacteroidetes*, *Fusobacteria*, and *Spirochaetes*. Similarities were detected between bacterial families and genera in both indigenous backyard and commercial chickens; however, the bacterial family *Bacillaceae* and genus *Anoxybacillus* were only observed in commercial chickens. Statistical tests performed to evaluate the alpha‐diversity and beta‐diversity metrics showed no significant difference in the fecal bacterial microbiota of indigenous backyard and commercial chickens, as indicated by the Wilcoxon rank‐sum (*p* = 0.94) and PERMANOVA tests (*p* = 0.26). This study highlights bacteria that may affect the growth, development, and health of indigenous and commercial poultry raised under various production systems, thereby providing vital insights for the development of effective poultry farm management practices.

## 1. Introduction

The gastrointestinal (GI) tract harbors a variety of microorganisms, including bacteria, viruses, and fungi, along with their collective genetic material, collectively known as the gut microbiome [1]. Research has shown that the gut microbiota plays a pivotal role in numerous essential host functions, including physiological, metabolic, immunological, digestive, and nutritional processes [2]. Feed conversion, nutrient absorption, and productivity are highly dependent on the gut flora and proper GI function of the host [3]. The gut microbiota composition in chickens undergoes dynamic changes over time, influenced by factors such as host genetic makeup, age, environmental conditions, and diet [4, 5]. Understanding the gut microbiota is essential, as this knowledge can be leveraged to combat antimicrobial resistance, enhance food quality and safety, and safeguard public health [6]. By examining the composition, functions, and interactions of the gut microbiota in animals, researchers can devise effective strategies to mitigate microbial hazards, curb antimicrobial resistance, and enhance the safety of food products for consumers [7, 8].

Poultry production systems vary across countries and economic groups, depending on local resources, infrastructure, and market demands. These differences can be reflected in the composition of the intestinal microbiome [9]. In the free‐range (backyard or scavenging) poultry production system, commonly adopted by rural smallholder farmers, indigenous backyard chickens roam freely and forage, consuming a diverse diet that includes flora, seeds, fruits, soil particles, microbes, insects, and worms, alongside the corn–soybean meal–based feed provided by humans [10, 11]. This production system is also proposed to improve chicken health, welfare, and gut microbial diversity [12, 13]. On the contrary, the gut microbiota composition of commercial chicken breeds raised in intensive production systems is significantly influenced by the controlled environments to which the chickens are subjected [14]. Even though the characterization of bacterial microbiota in various parts of the GI tract, including the oral pharyngeal, cecal, and fecal features, has recently garnered significant attention in chicken research [15], there is limited information on the gut microbiota profiles of indigenous backyard chickens raised in the free‐range production system.

Recent technological advancements have alleviated the challenges associated with culturing individual microorganisms. Instead, modern methods rely on analyzing bacterial community structure by detecting distinctive microbial DNA markers, such as 16S rRNA, isolated from community samples [16, 17]. Using these methods, researchers have discovered that 90% of the bacteria in the chicken GI tract belong to previously unknown species [18]. Metagenomics, a nonculture‐based technique, has enabled researchers to conduct in‐depth studies of microbial community composition across diverse habitats and sample types [19]. To investigate the gut microbiota of chickens, fecal samples have been used as a proxy because they can be collected noninvasively [20]. In addition, Stanley et al. [18] examined microbial interactions between the ceca, which are internal structures of the large intestine, and feces, finding that 88.55% of operational taxonomic units (OTUs) were shared between the two sites.

Gut microbiota profiling could enhance our understanding of the bacterial communities that influence the growth and development of indigenous backyard chickens. This knowledge can inform nutritional and management practices to improve intestinal health and overall performance. To investigate and characterize the fecal microbiota of indigenous backyard and commercial chickens raised in different production systems and to identify potentially economically significant bacterial taxa for the poultry industry, 16S rRNA metagenomic analysis was conducted.

## 2. Materials and Methods

### 2.1. Study Area and Data Collection

**FIGURE 1:**
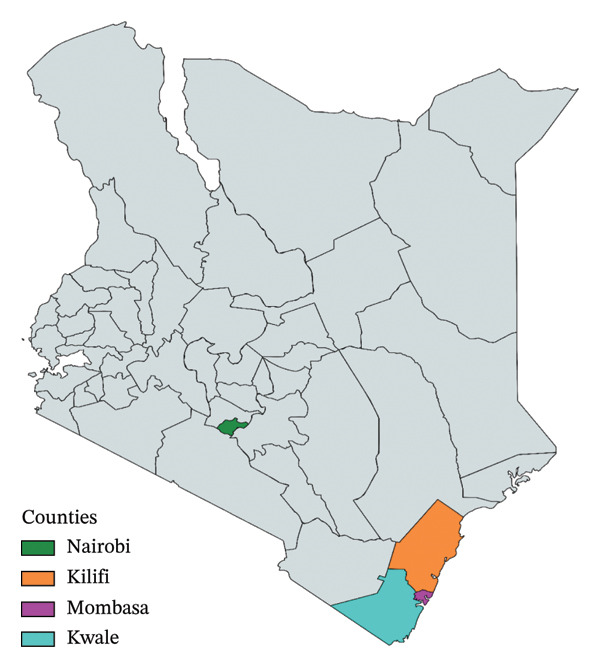
Sampling locations in Kenya.

**FIGURE 2:**
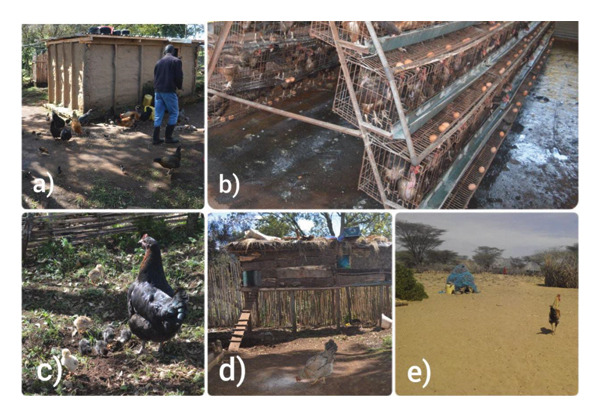
Chicken production systems in Kenya: (a) feed supplementation with maize in the semi‐intensive production system; (b) commercial layer chicken in an intensive production system; (c) indigenous backyard chickens scavenging for food; (d) semi‐intensive indigenous backyard chicken shelter; (e) makeshift free‐range indigenous backyard chicken shelter.

This study used archived samples collected in June 2018 from Nairobi, Kilifi, Kwale, and Mombasa counties (Figure 1). Kilifi, Kwale, and Mombasa were intentionally selected due to their high population density of indigenous backyard poultry. In these regions, rural smallholder farmers employ the free‐range and semi‐intensive chicken production systems. In contrast, the commercial chickens used as controls were sampled from farms in Nairobi that often employ intensive production systems (Figure 2).

Fecal swab samples from 18 asymptomatic indigenous backyard chickens and 13 commercial chickens that were previously preserved at −80°C were selected for this study.

### 2.2. DNA Extraction

Genomic DNA was extracted from the samples using the phenol–chloroform method [21]. It involved extracting genomic DNA from the samples using Tris‐EDTA‐saturated phenol:chloroform (1:1). 250 μL of the sample homogenate was then mixed with 250 μL of phenol:chloroform (1:1), vortexed for 10 s, and centrifuged at 14,000 × g for 3 min. The aqueous phase was then collected in a fresh tube, and an equal volume of chloroform was added. The mixture was vortexed and centrifuged at 14,000 × g for 3 min. The aqueous phase was again collected in a fresh tube, and three volumes of 95% ethanol/0.12 M sodium acetate were added. The mixture was then mixed by inversion, incubated on ice for 10 min, and centrifuged at 14,000 × g for 15 min at 4°C. The supernatant was then decanted, and 80% ethanol was added, corresponding to 2 volumes of the original sample. The samples were then incubated at room temperature for 10 min and centrifuged at 14,000 × g for 5 min. The supernatant was then decanted, and the pellet was dried in a SpeedVac (Thermo Electron Corporation) for 10 min at 37°C. Purified DNA was then hydrated with 100 μL of nuclease‐free water and stored at −80°C for long‐term use.

### 2.3. 16S rRNA V3‐V4 Region Amplification and Sequencing

The V3‐V4 hypervariable region of the 16S rRNA gene was amplified from genomic DNA using primers adapted from Kumar et al. [22]. PCR amplification was performed in 50 μL reaction mixtures using the NEBNext High‐Fidelity 2X PCR Master Mix (New England Biolabs, USA). The PCR reactions included an initial denaturation step at 98°C for 30 s, followed by 25 cycles of 98°C for 10 s, 62°C for 30 s, 72°C for 30 s, and a final extension step at 72°C for 5 min in the SureCycler 8800 Thermal Cycler (Agilent, USA). The PCR products were separated on a 1.5% agarose gel by electrophoresis and purified using a QIAquick gel extraction kit (QIAGEN, Germany). DNA quality and concentration were checked using a Quantus fluorometer (Promega, USA). The amplicons were then sent to a commercial sequencing facility (BGI Genomics, Shenzhen) for sequencing on the Illumina MiSeq platform.

### 2.4. Bioinformatics Analysis

The sequence data were processed in mothur v1.33.3 [23], where they were aligned to a curated 16S rRNA reference database and clustered into OTUs at a 97% sequence identity threshold to characterize overall bacterial community structure and identify dominant taxa. This OTU‐based workflow was selected to ensure comparability with previous 16S rRNA studies using the same pipeline and because OTU and amplicon sequence variant (ASV) approaches have been shown to yield broadly comparable beta‐diversity patterns and dominant taxa in microbiome studies [24].

The paired‐end reads underwent initial quality assessment with FastQC Version 0.11.9 [25] and then were joined into contigs, and a Phred quality threshold (*Q* ≥ 25) was applied for stringent quality control. Any contigs with ambiguous bases (N) and shorter than 200 base pairs were culled, while identical or duplicate sequences were merged. Sequences were then aligned to the SILVA database Version 132 [26]. Poorly aligned sequences were removed, and overhangs at both ends were trimmed so that they overlapped in the same region. Unique sequences were screened and further denoised based on a preclustered command, allowing up to 2 differences between sequences. Chimeric sequences were removed using the UCHIME program Version 4.2.40 [27]. The OTU‐based method was used for analysis, in which sequences were assigned to bins by taxonomy and clustered within each bin using a 0.03 cutoff.

OTU tables and other outputs from mothur were further analyzed in R (Version 4.4.2) using the *Phyloseq* [28] and *vegan* [29] packages and visualized with the *ggplot2* [30] package and the web‐based tool ImageGP [31]. Alpha (*α*) diversity indices, including ACE and Shannon, were calculated to assess the species richness and diversity of the sequences within the community. Beta (*β*) diversity analysis was performed to investigate the structural variation of microbial communities between indigenous backyard and commercial samples using the UniFrac distance metric [32], which was then visualized using the principal coordinate analysis (PCoA) [33]. Statistical analysis of alpha diversity was performed using the nonparametric Wilcoxon rank‐sum test. To investigate differences between the indigenous backyard and commercial chicken microbiota, a nonparametric permutational multivariate analysis of variance (PERMANOVA) was performed using the “*Adonis*” function of the *vegan* R package with 999 permutations. The statistical significance of all comparisons was assessed at a *p* value of 0.05.

## 3. Results

### 3.1. Sequencing Results

High‐throughput sequencing generated 1,992,189 sequences, corresponding to approximately 883 MB of data, from 31 chicken fecal samples. After read quality filtering, merging paired‐end reads, denoising, removing chimeras, and filtering low‐quality sequences, the average number of quality‐controlled sequences per sample was 44,815. For each location, the sequences were merged and OTUs were clustered at > 97% similarity using mothur (Table 1).

**TABLE 1:** Summary statistics of sequences analyzed including average OTU numbers detected.

Type	Location	Total sequences	Average sequences/sample	Average number of OTUs
Commercial	Nairobi	863,848	66,450	1179

Indigenous	Mombasa	375,893	62,649	1381
	Kwale	392,673	65,446	954
	Kilifi	359,775	59,963	890

### 3.2. Alpha Diversity and Beta Diversity Analysis

**FIGURE 3:**
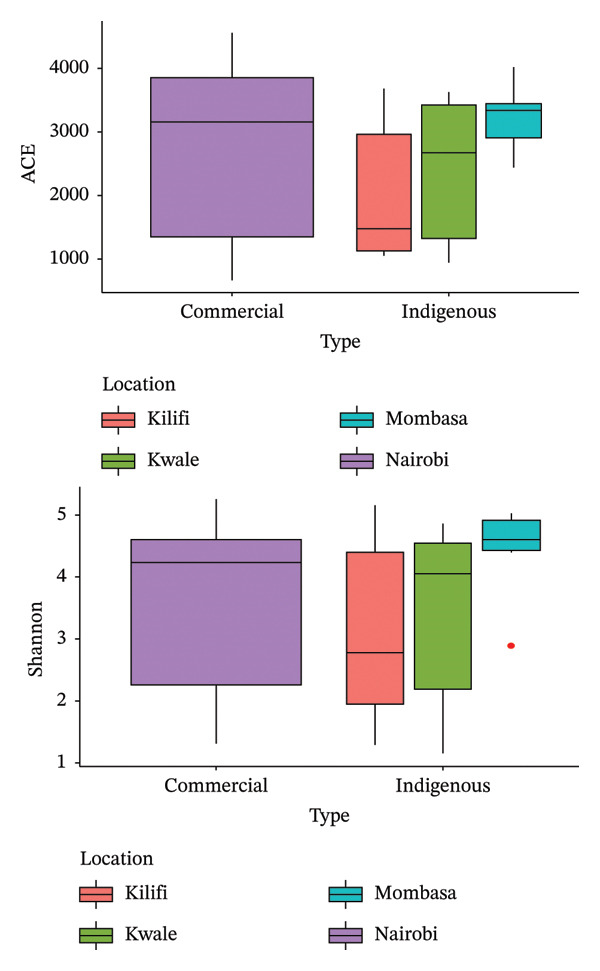
A comparison of the ACE and Shannon indices between the commercial and indigenous backyard chicken bacterial microbiota. Box plots show the quartiles, median, and extremities of the values.

The alpha diversity of the bacterial microbiota in indigenous backyard and commercial chickens was assessed using the ACE and Shannon indices to evaluate species richness and diversity. The ACE and Shannon indices were generally higher in commercial chickens than in indigenous backyard chickens. However, indigenous backyard chicken samples from Mombasa, a metropolitan area, had high values similar to those of commercial chickens from the Nairobi metropolitan area (Figure 3). These differences in species richness and diversity were, however, not statistically significant (*p* = 0.94), as evidenced by the Wilcoxon rank‐sum test.

**FIGURE 4:**
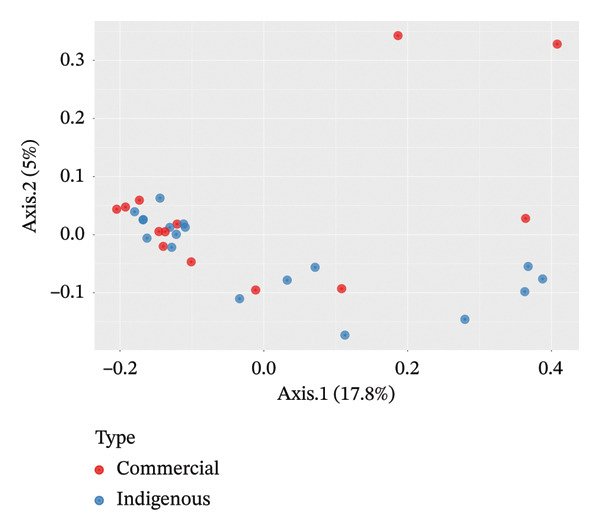
PCoA plot based on unweighted UniFrac distance matrix between the bacterial microbiota in indigenous backyard and commercial chicken.

**FIGURE 5:**
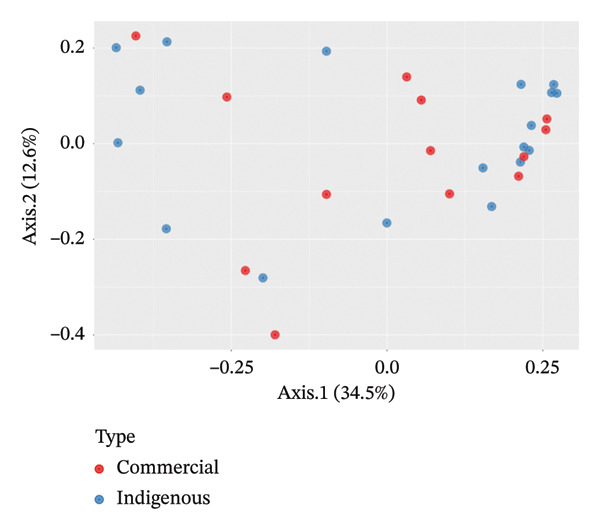
PCoA plot based on weighted UniFrac distance matrix between the bacterial microbiota in the indigenous backyard and commercial chicken.

Beta‐diversity analysis using unweighted and weighted UniFrac metrics was performed to assess distances among the bacterial microbiota of indigenous backyard and commercial chickens. In both plots, Axis 1 captured differences between the bacterial microbiota of indigenous backyard and commercial chickens, accounting for 17.8% and 34.5% of the variation, respectively. The bacterial microbiota profiles of indigenous backyard and commercial chickens showed no distinct separation. However, commercial chickens exhibited slightly higher diversity (Figures 4 and 5).

Using PERMANOVA, indigenous backyard and commercial chickens showed no statistically significant differences in bacterial microbiota composition (*p* = 0.26).

**FIGURE 6:**
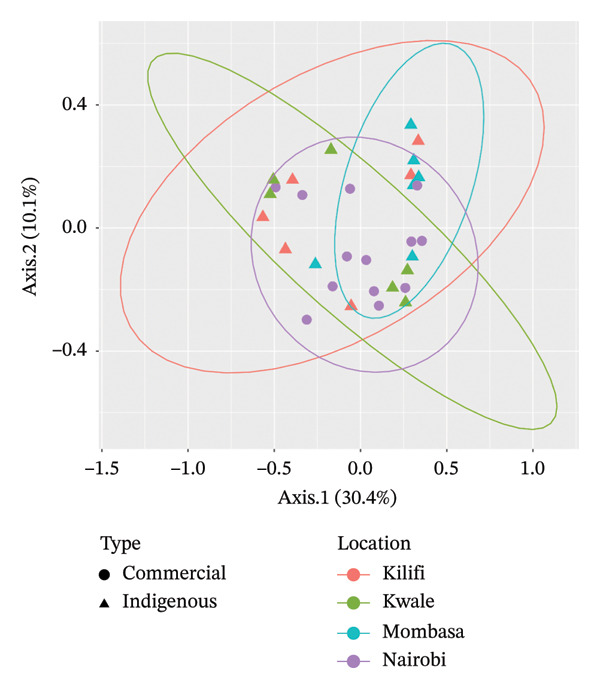
Common and unique bacterial microbiota among the indigenous backyard and commercial chickens in the four locations. A Venn diagram showing the overlaps.

Visualizing the OTU distribution using a Venn diagram further demonstrated that the fecal microbiota of all the chickens from the four locations did not show any divergence, and a greater variety of overlaps were shared by all the plotted groups (Figure 6).

### 3.3. Taxonomic Assignment

Taxonomic assignment was performed using an OTU‐based approach, clustering sequences with at least 97% sequence identity. The consensus taxonomy assignment identified 19 phyla, 34 classes, 71 orders, 137 families, and 238 genera.

#### 3.3.1. Taxonomy at the Phylum Level

**FIGURE 7:**
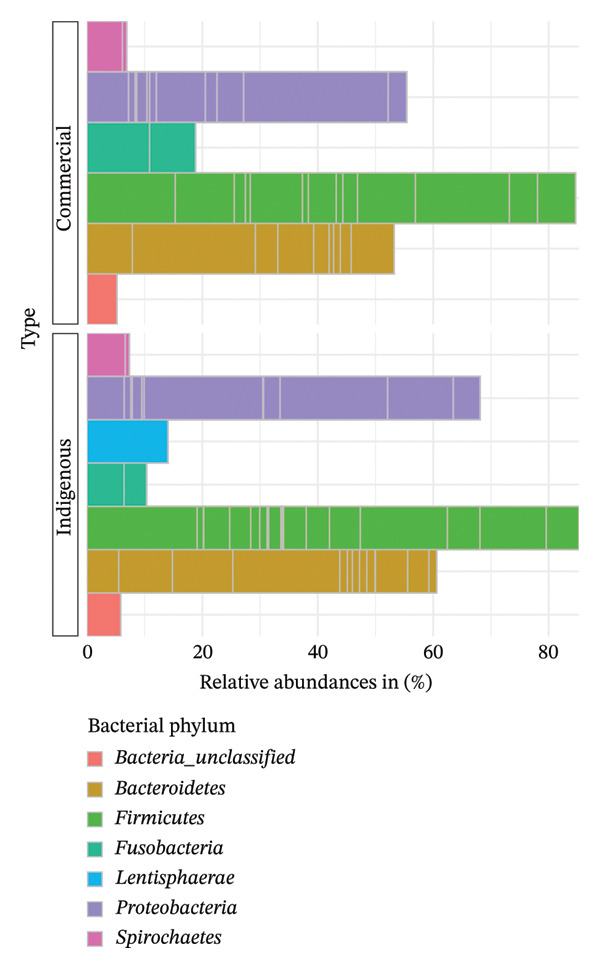
Taxonomic profile at the phylum level. A bar chart comparing the percentage relative abundances (> 5%) of bacteria present in indigenous backyard and commercial chickens.

The most abundant phyla in the indigenous backyard chicken were *Firmicutes*, *Proteobacteria*, *Bacteroidetes*, and *Lentisphaerae*, with abundances of 84%, 79%, 61%, and 13%, respectively. Similarly, the top three phyla remained dominant in commercial chicken, though abundances varied, with *Fusobacteria* (19%) as the fourth most abundant phylum (Figure 7).

**FIGURE 8:**
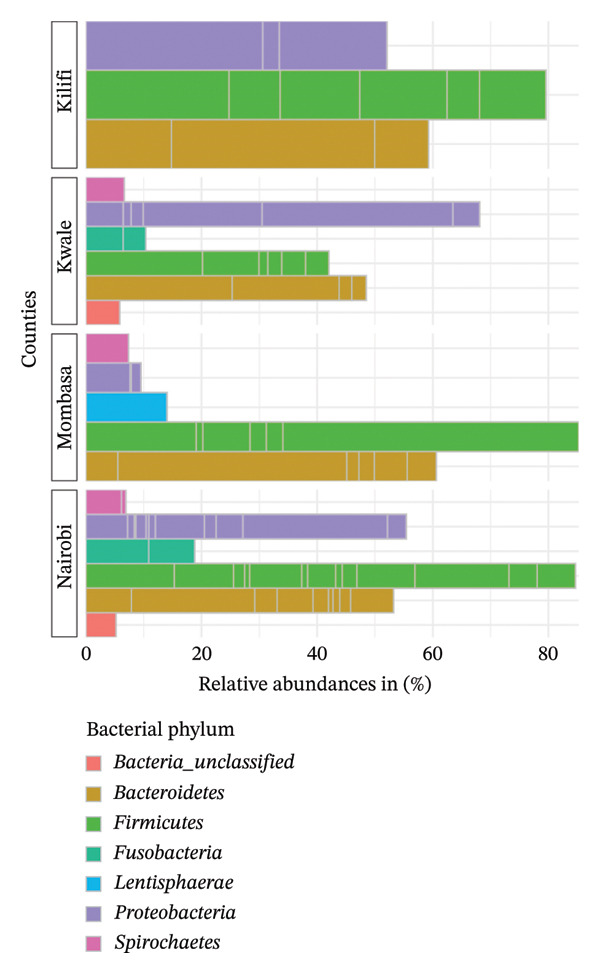
Taxonomic profile at the phylum level. A bar chart comparing the percentage relative abundances (> 5%) of bacteria present in indigenous backyard and commercial chickens in the respective counties.

However, the relative abundances of indigenous backyard chickens varied across counties (Figure 8). The most abundant phylum in Mombasa County was *Firmicutes* (84%), followed by *Bacteroidetes* (60%) and *Lentisphaerae* (15%). In Kilifi County, *Firmicutes* (80%) and *Bacteroidetes* (59%) remain the predominant phyla, followed by *Proteobacteria* (52%). However, in Kwale County, *Proteobacteria* (68%) dominate, followed by *Bacteroidetes* (49%) and finally *Firmicutes* (42%). Overall, the three phyla *Firmicutes* (45%), *Bacteroidetes* (25%), and *Proteobacteria* (22%) collectively represented 92% of the total bacterial count.

#### 3.3.2. Taxonomy at the Family Level

**FIGURE 9:**
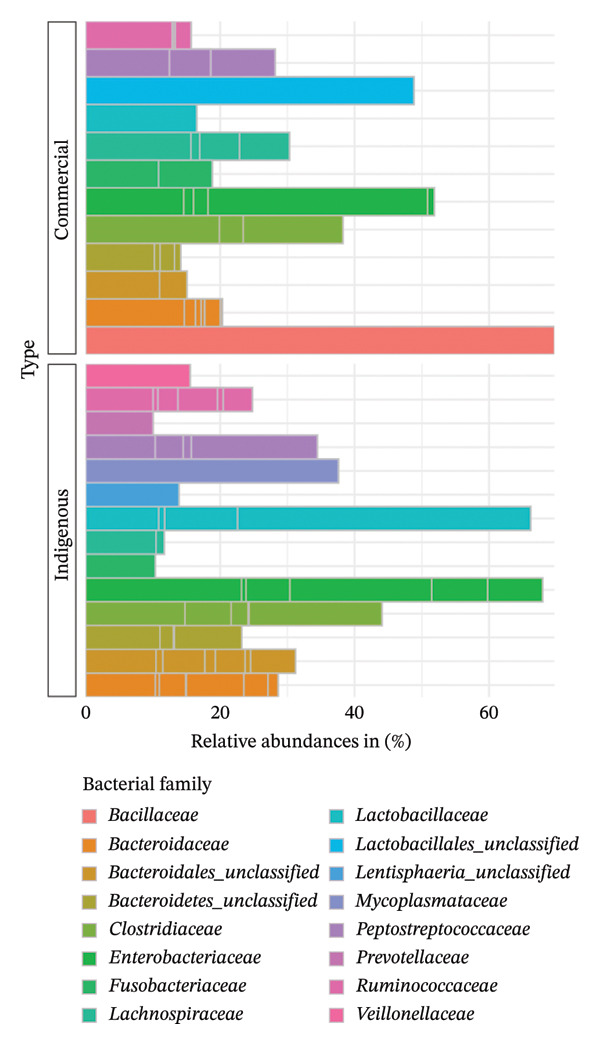
Taxonomic profile at the family level. A bar chart comparing the percentage relative abundances (> 8%) of bacteria present in indigenous backyard and commercial chickens.

A general classification at the family level revealed *Bacillaceae, Enterobacteriaceae*, and *Lactobacillaceae* as the most dominant bacterial families (Figure 9). In the indigenous backyard chicken, *Enterobacteriaceae* (69%) were the most abundant, followed closely by *Lactobacillaceae* (68%).

**FIGURE 10:**
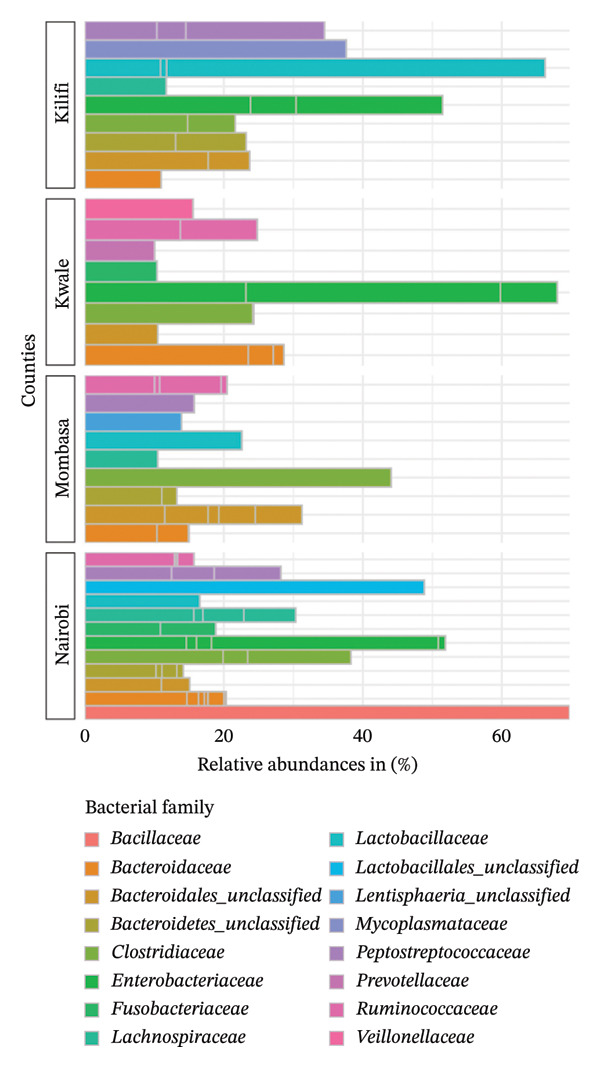
Taxonomic profile at the family level. A bar chart comparing the percentage relative abundances (> 8%) of bacteria present in indigenous backyard and commercial chickens in the respective counties.

At the family level, the most dominant bacterial family differed between indigenous backyard chickens and commercial chickens, as well as between the counties (Figure 10). In Kwale County, the *Enterobacteriaceae* family was the most dominant, accounting for 64%, whereas in Kilifi County, the *Lactobacillaceae* family was the most dominant, at 63%. In Mombasa County, the *Clostridiaceae* was the most dominant family, accounting for 43%. In contrast, the *Bacillaceae* family was only found in commercial chickens and was the most dominant there.

#### 3.3.3. Taxonomy at the Genus Level

**FIGURE 11:**
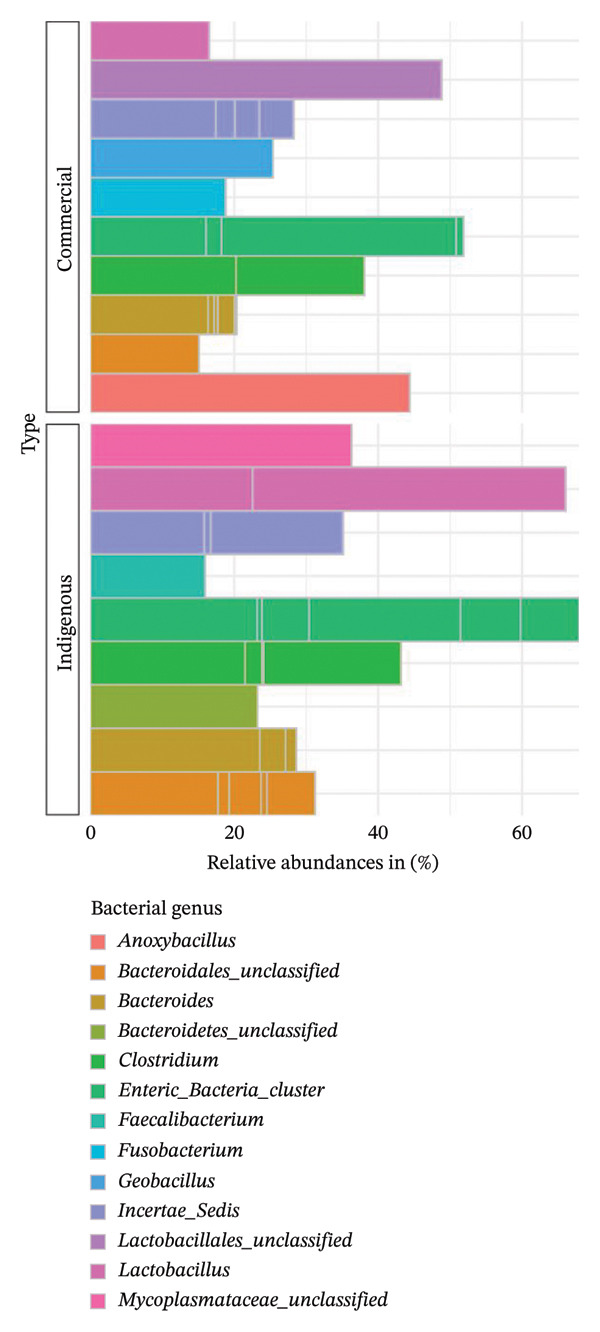
Taxonomic profile at the genus level. A bar chart comparing the percentage relative abundances (> 10%) of bacteria present in indigenous backyard and commercial chickens.

At the genus level, the most abundant bacteria in both indigenous backyard and commercial chickens were *Enteric_bacteria_cluster* belonging to the *Enterobacteriaceae* family and the *Proteobacteria* phylum. This was followed by *Lactobacillus* of the *Lactobacillaceae* family and the *Firmicutes* phylum (Figure 11).

**FIGURE 12:**
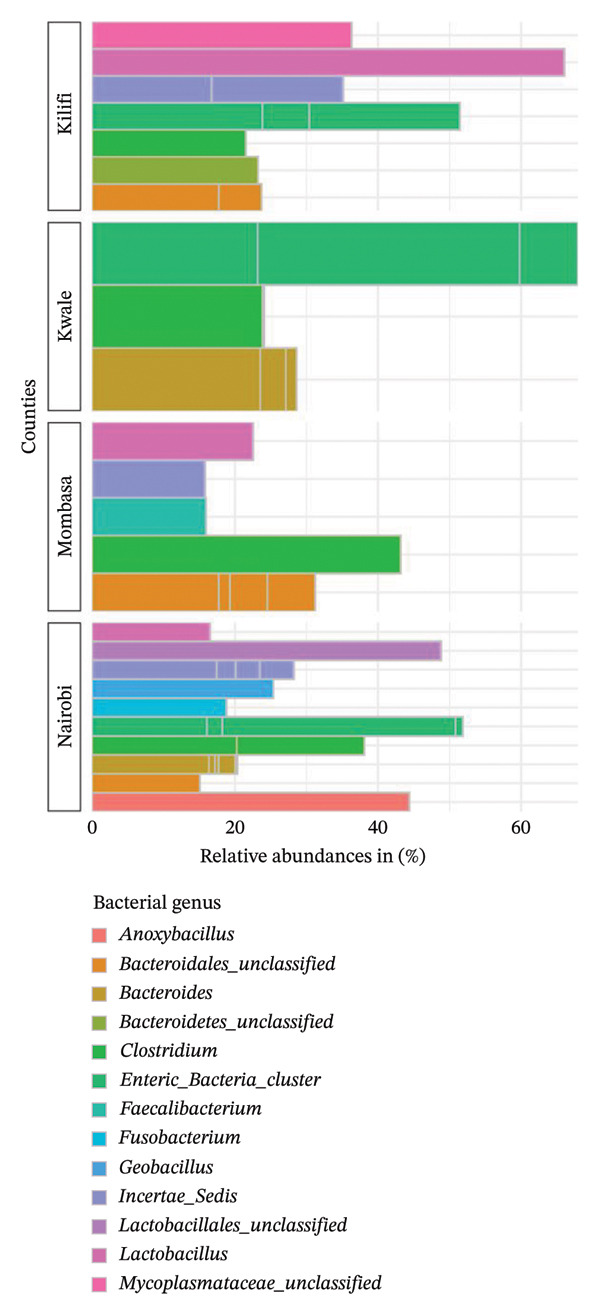
Taxonomic profile at the genus level. A bar chart comparing the percentage relative abundances (> 10%) of bacteria present in indigenous backyard and commercial chickens in the respective counties.

The genus *Enteric_bacteria_cluster* was the most abundant in both indigenous backyard and commercial chicken at 69% and 52%, respectively. However, the genus *Lactobacillus* was second in abundance at 68% in indigenous backyard chickens, unlike in commercial chickens, where the genus *Lactobacillales_unclassified* was second at 49%. Additionally, the genus *Anoxybacillus* was detected only in commercial chicken samples at 45%. Comparing the bacterial microbiota in the various coastal counties, the genus *Lactobacillus* was the most predominant in Kilifi County (65%), whereas *Enteric_bacteria_cluster* and *Clostridium* were predominant in Kwale (68%) and Mombasa (43%) counties, respectively (Figure 12).

## 4. Discussion

Free‐range (backyard or scavenging) and intensive poultry production systems apply different feeding and biosecurity practices, which can impact the poultry gut microbiota differently. Chickens raised under these production systems are believed to have unique gut microbiota profiles due to the varying farm management conditions they experience. These include housing, environmental factors, diet, and biosecurity measures, which influence the diversity and composition of the gut microbiota, ultimately affecting poultry productive performance and overall health [34, 35].

The GI tract harbors a diverse population of microorganisms that have been detected in various studies conducted on indigenous and commercial chickens [36–38]. Recently, our research group conducted a metagenomic analysis of cloacal and oropharyngeal swabs to examine the bacterial microbiota in various poultry species, including indigenous backyard chickens [8]. However, a gap remains in our understanding of the bacterial microbiota of poultry species raised in different production systems in Kenya, particularly for indigenous backyard and commercial chickens. In this study, we successfully identified and compared the bacterial composition, relative abundances, and taxonomy of the fecal microbiota of Kenyan chickens raised in both free‐range and intensive production systems, using targeted PCR amplification and high‐throughput 16S rRNA sequencing data.

The Wilcoxon rank‐sum and PERMANOVA statistical tests revealed no significant difference in bacterial composition between indigenous backyard and commercial chickens. This could be due to an observed overlap between production systems in some farms, particularly in regions close to cities and townships, such as Mombasa, where free‐range, semi‐intensive, and intensive production system practices are implemented, including the provision of food supplementation and proper housing. Despite scavenging for food, these indigenous backyard chickens are often provided with supplemental feeds when near the homestead and share housing with commercial chickens in separate compartments [39]. Due to rising feed costs, most commercial chickens raised in urban centers are now supplemented with food leftovers and maize mill waste, similar to indigenous backyard chickens, in addition to processed feed [35]. Furthermore, farms implementing these production systems generally rely on the same water sources, such as boreholes and water vendors, for poultry water [39, 40]. These practices fail to clearly distinguish between the feed and water taken by commercial chickens and indigenous backyard chickens.

The most prevalent phyla in both indigenous backyard and commercial chickens were *Firmicutes*, *Proteobacteria*, and *Bacteroidetes*, consistent with the findings of Panyako et al. [8], who used shotgun metagenomic sequence data from poultry cloacal samples in Kenya. However, some differences were also observed in the bacterial microbiota of indigenous backyard and commercial chickens. The unique *Lentisphaerae* bacterial phylum, which was found only in indigenous backyard chickens from Mombasa County, is widely distributed across various environments. It has been detected in seawater [41, 42], sediments [43], anaerobic sludge [44], landfill leachate [45], and the guts of both animals and humans [46, 47].

*Lentisphaerae* bacteria are predicted to be closely associated with biogeochemical cycles and bioremediation and are suggested to play a significant role in directing fundamental pathways in elemental and nutrient cycling [48, 49].

Furthermore, these bacteria may be involved in polysaccharide degradation [50] and in various health issues, including type 2 diabetes mellitus (T2DM) in humans [51], inflammation in the terminal ileum [52], and autoimmune hepatitis [53]. It has been suggested that chronic systemic inflammation and T2DM can be improved through bacterial translocation. Therefore, it is important to develop feeds that enhance the presence of short‐chain fatty acid–producing bacteria while reducing the number of Gram‐negative endotoxin‐producing bacteria [51].

The dominance of the family *Enterobacteriaceae* and, equally, the genus *Enteric_bacteria_cluster* among indigenous backyard chickens is a point of concern. *Enterobacteriaceae* species are commonly found in the digestive systems of animals. While most are harmless or beneficial, some genera, such as *Enterobacter*, *Escherichia*, *Proteus*, and *Salmonella*, are medically significant and warrant special attention [54]. Fecal contamination of animal products by bacteria such as *Salmonella*, *Escherichia coli*, *Klebsiella*, and *Proteus* is a known major cause of foodborne illnesses [55]. Other bacteria in this family contribute to food spoilage, affecting products such as meat, eggs, milk, fish, seafood, and dairy, resulting in significant economic losses [56]. Recent reports have documented foodborne outbreaks associated with certain enterobacterial strains exhibiting “hybrid” virulence traits. This information has been documented by several studies [57–59]. Given the widespread presence of enterobacteria in food and the associated hygiene and sanitation challenges, developing nations such as Kenya are particularly vulnerable to serious health issues caused by foodborne illnesses from these bacteria.

It has been observed that the use of antimicrobial drugs in food animals, such as chickens, has resulted in the transfer of resistant pathogen strains to humans through food consumption [8, 60]. The transfer of resistant pathogen strains and their genes into nonpathogenic commensal microflora can occur in the digestive tract of many food animals, including chickens [61]. Antimicrobial resistance genes found in bacteria of the family *Enterobacteriaceae* are a clear indication of the presence of resistant bacterial strains in the community [55, 62]. Studies have reported that members of the *Enterobacteriaceae* family, such as *E. coli*, *Klebsiella*, *Proteus*, and *Salmonella*, have developed antimicrobial resistance [61, 63]. This highlights the need for further research and action to prevent the spread of these resistant bacterial strains to other hosts, including humans. It is essential to pay attention to indigenous backyard chickens raised under a free‐range production system. These chickens may serve as crucial environmental indicators of multidrug resistance. Indigenous backyard chickens tend to be more disease‐tolerant and may act as spreaders and long‐term reservoirs for medically threatening pathogens that carry resistance genes. This role may be more significant than previously thought, making it essential to monitor their health and well‐being closely [64].

While no statistically significant differences in alpha‐ and beta‐diversity metrics were observed between indigenous backyard and commercial chickens, the overall community profiles suggest a largely shared core microbiota. Nevertheless, minor but noticeable differences in the bacterial microbiota between the two groups were identified. This highlights that the gut microbiome is often dynamic and that the bacterial microbiota can vary across poultry production systems. However, differences in host genetics between indigenous backyard and commercial chickens can also play a significant role in shaping the gut microbiome [65]. Additionally, the use of archived samples in this study may have limited the detection of additional bacterial taxa compared with fresh samples. Given our sample size and analyses, the study may lack the power to detect small but potentially meaningful differences in bacterial composition. Therefore, the lack of statistical significance should not be interpreted as definitive evidence of complete biological equivalence among the poultry types; further studies are warranted to explore this relationship more thoroughly. Nevertheless, this study provides valuable information that can serve as a step toward understanding the gut microbiomes of indigenous backyard chickens and commercial poultry raised in various production systems across many developing nations and how those production systems affect the poultry microbiome and possibly the humans who consume them.

## 5. Conclusion

Our study identified similarities and differences in the bacterial microbiota of indigenous backyard and commercial chickens raised under free‐range and intensive poultry production systems. The most prevalent phyla in indigenous backyard and commercial chickens were *Firmicutes*, *Proteobacteria*, *Bacteroidetes*, *Fusobacteria*, and *Spirochaetes*, respectively. However, no significant differences in species richness and diversity were observed. The dominance of the *Enterobacteriaceae* family in indigenous backyard chickens, which are well known for their resistance to antibiotics, raises concerns about antimicrobial use in free‐range (backyard or scavenging) poultry production systems, particularly among poor rural smallholder farmers who possess little to no farm biosecurity knowledge or expertise. Although genomic surveillance through metagenomic analysis has enabled the effective detection and identification of these foodborne pathogens in food‐producing animals, thereby improving food safety, antimicrobial use in food‐producing animals, such as indigenous backyard chickens, should be closely monitored. This is necessary to prevent the growth and transmission of antimicrobial‐resistant bacteria and their determinants from food animals to humans, which could pose a serious public health threat. This is a crucial step toward antimicrobial stewardship.

## Funding

This work was supported by the Sino‐Africa Joint Research Center, Chinese Academy of Sciences (CAS) (SAJC201611), Animal Branch of the Germplasm Bank of Wild Species, CAS (Large Research Infrastructure Funding), and Bureau of Science and Technology of Yunnan Province.

Open access publishing facilitated by The University of Queensland, as part of the Wiley ‐ The University of Queensland agreement via the Council of Australasian University Librarians.

## Ethics Statement

A “no objection” permit was obtained from the Ministry of Agriculture, Livestock, and Fisheries, Directorate of Veterinary Srvices, Kenya (RES/POL/VOL.XXVII/162), to sample indigenous backyard and commercial chickens and carry out the research.

## Conflicts of Interest

The authors declare no conflicts of interest.

## Data Availability

The sequencing data that support the findings of this study are openly available in the National Center for Biotechnology Information (NCBI) database under the BioProject PRJNA1293832.

## References

[1] Ahn J. S. , Lkhagva E. , Jung S. , Kim H. J. , Chung H. J. , and Hong S. T. , Fecal Microbiome Does Not Represent Whole Gut Microbiome, Cellular Microbiology. (2023) 2023, 1–14, 10.1155/2023/6868417.

[2] Yeoman C. J. , Chia N. , Jeraldo P. , Sipos M. , Goldenfeld N. D. , and White B. A. , The Microbiome of the Chicken Gastrointestinal Tract, Animal Health Research Reviews. (2012) 13, no. 1, 89–99, 10.1017/S1466252312000138, 2-s2.0-84875807802. 22853945

[3] Day J. M. , Oakley B. B. , Seal B. S. , and Zsak L. , Comparative Analysis of the Intestinal Bacterial and RNA Viral Communities From Sentinel Birds Placed on Selected Broiler Chicken Farms, PLoS One. (2015) 10, no. 1, 10.1371/journal.pone.0117210, 2-s2.0-84922463426. PMC4311960 25635690

[4] Xu Z. R. , Hu C. H. , Xia M. S. , Zhan X. A. , and Wang M. Q. , Effects of Dietary Fructooligosaccharide on Digestive Enzyme Activities, Intestinal Microflora and Morphology of Male Broilers, Poultry Science. (2003) 82, no. 6, 1030–1036, 10.1093/ps/82.6.1030, 2-s2.0-0042708599. 12817461

[5] Yadav S. and Jha R. , Strategies to Modulate the Intestinal Microbiota and Their Effects on Nutrient Utilization, Performance, and Health of Poultry, Journal of Animal Science and Biotechnology. (2019) 10, 1–11, 10.1186/s40104-018-0310-9, 2-s2.0-85059988435. 30651986 PMC6332572

[6] Odey T. O. J. , Tanimowo W. O. , Afolabi K. O. , Jahid I. K. , and Reuben R. C. , Antimicrobial Use and Resistance in Food Animal Production: Food Safety and Associated Concerns in Sub-Saharan Africa, International Microbiology. (2024) 27, no. 1, 1–23, 10.1007/s10123-023-00462-x. 38055165 PMC10830768

[7] Nelson D. W. , Moore J. E. , and Rao J. R. , Antimicrobial Resistance (AMR): Significance to Food Quality and Safety, Food quality and safety. (2019) 3, no. 1, 15–22, 10.1093/fqsafe/fyz003, 2-s2.0-85065637398.

[8] Panyako P. M. , Ommeh S. C. , Kuria S. N. et al., Metagenomic Characterization of Poultry Cloacal and Oropharyngeal Swabs in Kenya Reveals Bacterial Pathogens and Their Antimicrobial Resistance Genes, International Journal of Microbiology. (2024) 2024, no. 1, 10.1155/2024/8054338. PMC10876313 38374958

[9] McKenna A. , Ijaz U. Z. , Kelly C. et al., Impact of Industrial Production System Parameters on Chicken Microbiomes: Mechanisms to Improve Performance and Reduce Campylobacter, Microbiome. (2020) 8, no. 1, 10.1186/S40168-020-00908-8. PMC7488076 32907634

[10] Magothe T. M. , Okeno T. O. , Muhuyi W. B. , and Kahi A. K. , Indigenous Chicken Production in Kenya: I. Current Status, World’s Poultry Science Journal. (2012) 68, no. 1, 119–132, 10.1017/s0043933912000128, 2-s2.0-84857549573.

[11] Nzioka S. M. , Mungube E. O. , Mwangi M. D. , Muhammed L. , and Wambua J. M. , The Quantity and Quality of Feed Available to Indigenous Chickens Under the Scavenging System in Semi-Arid Eastern Kenya, East African Agricultural and Forestry Journal. (2017) 82, no. 1, 57–69, 10.1080/00128325.2016.1253324.

[12] Meng L. , Mao P. , Guo Q. , and Tian X. , Evaluation of Meat and Egg Traits of Beijing-You Chickens Rotationally Grazing on Chicory Pasture in a Chestnut Forest, Brazilian Journal of Poultry Science. (2016) 18, 1–6, 10.1590/1806-9061-2015-0081, 2-s2.0-84987624218.

[13] Taylor P. S. , Hemsworth P. H. , Groves P. J. , Gebhardt-Henrich S. G. , and Rault J.-L. , Frequent Range Visits Further From the Shed Relate Positively to Free-Range Broiler Chicken Welfare, Animal. (2020) 14, no. 1, 138–149, 10.1017/S1751731119001514, 2-s2.0-85068588411. 31280755

[14] Volf J. , Polansky O. , Varmuzova K. et al., Transient and Prolonged Response of Chicken Cecum Mucosa to Colonization With Different Gut Microbiota, PLoS One. (2016) 11, no. 9, 10.1371/journal.pone.016393. PMC5042506 27685470

[15] Shang Y. , Kumar S. , Oakley B. , and Kim W. K. , Chicken Gut Microbiota: Importance and Detection Technology, Frontiers in Veterinary Science. (2018) 5, 10.3389/fvets.2018.00254, 2-s2.0-85055813127. PMC6206279 30406117

[16] Giraffa G. and Neviani E. , DNA-Based, Culture-Independent Strategies for Evaluating Microbial Communities in Food-Associated Ecosystems, International Journal of Food Microbiology. (2001) 67, no. 1-2, 19–34, 10.1016/S0168-1605(01)00445-7, 2-s2.0-0035919814. 11482566

[17] Kumar S. S. and Ghosh A. R. , Assessment of Bacterial Viability: A Comprehensive Review on Recent Advances and Challenges, Microbiology. (2019) 165, no. 6, 593–610, 10.1099/mic.0.000786, 2-s2.0-85067105982. 30843781

[18] Stanley D. , Hughes R. J. , and Moore R. J. , Microbiota of the Chicken Gastrointestinal Tract: Influence on Health, Productivity and Disease, Applied Microbiology and Biotechnology. (2014) 98, no. 10, 4301–4310, 10.1007/s00253-014-5646-2, 2-s2.0-84900834533. 24643736

[19] Hilton S. K. , Castro-Nallar E. , Pérez-Losada M. et al., Metataxonomic and Metagenomic Approaches vs. Culture-Based Techniques for Clinical Pathology, Frontiers in Microbiology. (2016) 7, 10.3389/fmicb.2016.00484, 2-s2.0-84966350083. PMC4823605 27092134

[20] Yan W. , Sun C. , Zheng J. et al., Efficacy of Fecal Sampling as a Gut Proxy in the Study of Chicken Gut Microbiota, Frontiers in Microbiology. (2019) 10, 10.3389/fmicb.2019.02126, 2-s2.0-85072898925. PMC6753641 31572332

[21] Sambrook J. and Russell D. W. , Purification of Nucleic Acids by Extraction With Phenol:Chloroform, CSH Protocols. (2006) 2006, no. 1, 10.1101/pdb.prot4455. 22485786

[22] Kumar S. , Chen C. , Indugu N. et al., Effect of Antibiotic Withdrawal in Feed on Chicken Gut Microbial Dynamics, Immunity, Growth Performance and Prevalence of Foodborne Pathogens, PLoS One. (2018) 13, no. 2, 10.1371/journal.pone.0192450, 2-s2.0-85042152101. PMC5812630 29444134

[23] Schloss P. , Westcott S. , Ryabin T. et al., Introducing Mothur: Open-Source, Platform-Independent, Community-Supported Software for Describing and Comparing Microbial Communities, Applied and Environmental Microbiology. (2009) 75, no. 23, 7537–7541, 10.1128/AEM.01541-09, 2-s2.0-72949107142. 19801464 PMC2786419

[24] López-García A. , Pineda-Quiroga C. , Atxaerandio R. et al., Comparison of Mothur and QIIME for the Analysis of Rumen Microbiota Composition Based on 16S rRNA Amplicon Sequences, Frontiers in Microbiology. (2018) 9, 10.3389/fmicb.2018.03010, 2-s2.0-85058274660. PMC6300507 30619117

[25] Andrews S. , FastQC: A Quality Control Tool for High Throughput Sequence Data, 2010, http://www.bioinformatics.babraham.ac.uk/projects/fastqc/.

[26] Yilmaz P. , Parfrey L. W. , Yarza P. et al., The SILVA and “All-Species Living Tree Project (LTP)” Taxonomic Frameworks, Nucleic Acids Research. (2014) 42, D643–D648, 10.1093/NAR/GKT1209, 2-s2.0-84891813947. 24293649 PMC3965112

[27] Edgar R. C. , Haas B. J. , Clemente J. C. , Quince C. , and Knight R. , UCHIME Improves Sensitivity and Speed of Chimera Detection, Bioinformatics. (2011) 27, no. 16, 2194–2200, 10.1093/bioinformatics/btr381, 2-s2.0-79961181125. 21700674 PMC3150044

[28] McMurdie P. J. and Holmes S. , *Phyloseq*: An R Package for Reproducible Interactive Analysis and Graphics of Microbiome Census Data, PLoS One. (2013) 8, no. 4, 10.1371/JOURNAL.PONE.0061217, 2-s2.0-84876427223. PMC3632530 23630581

[29] Dixon P. , Computer Program Review VEGAN, a Package of R Functions for Community Ecology, Journal of Vegetation Science. (2003) 14, no. 6, 927–930, http://cran.r-project.org, 10.1111/j.1654-1103.2003.tb02228.x, 2-s2.0-1142306112.

[30] Wickham H. , Getting Started With ggplot2, ggplot2: Elegant Graphics for Data Analysis, 2016, Springer International Publishing, Cham, Germany, 11–31, 10.1007/978-3-319-24277-4_2.

[31] Chen T. , Liu Y.-X. , Huang L. , and Huang L. , ImageGP: An Easy-to-Use Data Visualization Web Server for Scientific Researchers, IMeta. (2022) 1, no. 1, 10.1002/IMT2.5. PMC10989750 38867732

[32] Lozupone C. and Knight R. , Unifrac: A New Phylogenetic Method for Comparing Microbial Communities, Applied and Environmental Microbiology. (2005) 71, no. 12, 8228–8235, 10.1128/AEM.71.12.8228-8235.2005, 2-s2.0-29144464937. 16332807 PMC1317376

[33] Ramette A. , Multivariate Analyses in Microbial Ecology, FEMS Microbiology Ecology. (2007) 62, no. 2, 142–160, 10.1111/J.1574-6941.2007.00375.X, 2-s2.0-35348933745. 17892477 PMC2121141

[34] Chen S. , Xiang H. , Zhang H. et al., Rearing System Causes Changes of Behavior, Microbiome, and Gene Expression of Chickens, Poultry Science. (2019) 98, no. 9, 3365–3376, 10.3382/ps/pez140, 2-s2.0-85071702304. 30916350

[35] Ogali I. N. , Okumu P. O. , Mungube E. O. et al., Genomic and Pathogenic Characteristics of Virulent Newcastle Disease Virus Isolated From Chicken in Live Bird Markets and Backyard Flocks in Kenya, International Journal of Microbiology. (2020) 2020, no. 1, 4705768–11, 10.1155/2020/4705768. 32908524 PMC7450340

[36] Al-Marzooqi W. , Al-Maskari Z. A. , Al-Kharousi K. , Johnson E. H. , and El Tahir Y. , Diversity of Intestinal Bacterial Microbiota of Indigenous and Commercial Strains of Chickens Using 16S rDNA-Based Analysis, Animals. (2020) 10, no. 3, 10.3390/ani10030391. PMC7143395 32121097

[37] Yadav S. , Caliboso K. D. , Nanquil J. E. et al., Cecal Microbiome Profile of Hawaiian Feral Chickens and Pasture-Raised Broiler (Commercial) Chickens Determined Using 16S rRNA Amplicon Sequencing, Poultry Science. (2021) 100, no. 7, 10.1016/j.psj.2021.101181. PMC8182230 34091350

[38] Adenaike A. S. , Akpan U. , Awopejo O. O. et al., Characterization of the Cecal Microbiome Composition of Nigerian Indigenous Chickens, Tropical Animal Health and Production. (2022) 54, no. 4, 10.1007/s11250-022-03191-x. 35687206

[39] Ochieng J. , Owuor G. , and Bebe B. O. , Management Practices and Challenges in Smallholder Indigenous Chicken Production in Western Kenya, Journal of Agriculture and Rural Development in the Tropics and Subtropics. (2013) 114, no. 1, 51–58, http://nbn-resolving.de/urn:nbn:de:hebis:34-2013030542607.

[40] Onono J. O. , Alarcon P. , Karani M. et al., Identification of Production Challenges and Benefits Using Value Chain Mapping of Egg Food Systems in Nairobi, Kenya, Agricultural Systems. (2018) 159, 1–8, 10.1016/J.AGSY.2017.10.001, 2-s2.0-85030987807. 31007360 PMC6472295

[41] Cho J. C. , Vergin K. L. , Morris R. M. , and Giovannoni S. J. , *Lentisphaera araneosa* Gen. nov., sp. nov, a Transparent Exopolymer Producing Marine Bacterium, and the Description of a Novel Bacterial Phylum, *Lentisphaerae* , Environmental Microbiology. (2004) 6, no. 6, 611–621, 10.1111/J.1462-2920.2004.00614.X, 2-s2.0-2642554918. 15142250

[42] Choi A. , Yang S. J. , Rhee K. H. , and Cho J. C. , *Lentisphaera marina* sp. nov., and Emended Description of the Genus *Lentisphaera* , International Journal of Systematic and Evolutionary Microbiology. (2013) 63, 1540–1544, 10.1099/IJS.0.046433-0, 2-s2.0-84876098205. 22888188

[43] Polymenakou P. N. , Lampadariou N. , Mandalakis M. , and Tselepides A. , Phylogenetic Diversity of Sediment Bacteria From the Southern Cretan Margin, Eastern Mediterranean Sea, Systematic & Applied Microbiology. (2009) 32, no. 1, 17–26, 10.1016/J.SYAPM.2008.09.006, 2-s2.0-58149484940. 19058941

[44] Qiu Y. L. , Muramatsu M. , Hanada S. , Kamagata Y. , Guo R. B. , and Sekiguchi Y. , *Oligosphaera ethanolica* Gen. nov., sp. nov., an Anaerobic, Carbohydrate-Fermenting Bacterium Isolated From Methanogenic Sludge, and Description of Oligosphaeria Classis nov. in the Phylum *Lentisphaerae* , International Journal of Systematic and Evolutionary Microbiology. (2013) 63, 533–539, 10.1099/IJS.0.039545-0, 2-s2.0-84874033089. 22523166

[45] Limam R. D. , Bouchez T. , Chouari R. et al., Detection of WWE2-Related *Lentisphaerae* by 16S rRNA Gene Sequencing and Fluorescence In Situ Hybridization in Landfill Leachate, Canadian Journal of Microbiology. (2010) 56, no. 10, 846–852, 10.1139/W10-065, 2-s2.0-77958560751. 20962908

[46] Liu Y. , Song X. , Zhou H. et al., Gut Microbiome Associates With Lipid-Lowering Effect of Rosuvastatin In Vivo, Frontiers in Microbiology. (2018) 9, 10.3389/FMICB.2018.00530, 2-s2.0-85044385044. PMC5874287 29623075

[47] Peng S. , Yin J. , Liu X. et al., First Insights Into the Microbial Diversity in the Omasum and Reticulum of Bovine Using Illumina Sequencing, Journal of Applied Genetics. (2015) 56, no. 3, 393–401, 10.1007/S13353-014-0258-1, 2-s2.0-84939650947. 25604266 PMC4543427

[48] Jiang X. , Dang H. , and Jiao N. , Ubiquity and Diversity of Heterotrophic Bacterial nasA genes in Diverse Marine Environments, PLoS One. (2015) 10, no. 2, 10.1371/JOURNAL.PONE.0117473, 2-s2.0-84922349751. PMC4315400 25647610

[49] Das R. and Kazy S. K. , Microbial Diversity, Community Composition and Metabolic Potential in Hydrocarbon Contaminated Oily Sludge: Prospects for in Situ Bioremediation, Environmental Science and Pollution Research. (2014) 21, no. 12, 7369–7389, 10.1007/S11356-014-2640-2, 2-s2.0-84902532665. 24682711

[50] Yan L. , Gao Y. , Wang Y. et al., Diversity of a Mesophilic Lignocellulolytic Microbial Consortium Which is Useful for Enhancement of Biogas Production, Bioresource Technology. (2012) 111, 49–54, 10.1016/J.BIORTECH.2012.01.173, 2-s2.0-84858753321. 22365718

[51] Maskarinec G. , Raquinio P. , Kristal B. S. et al., The Gut Microbiome and Type 2 Diabetes Status in the Multiethnic Cohort, PLoS One. (2021) 16, no. 6, 10.1371/JOURNAL.PONE.0250855. PMC8221508 34161346

[52] Fan H. N. , Zhu P. , Lu Y. M. et al., Mild Changes in the Mucosal Microbiome During Terminal Ileum Inflammation, Microbial Pathogenesis. (2020) 142, 10.1016/j.micpath.2020.104104. 32120004

[53] Lou J. , Jiang Y. , Rao B. et al., Fecal Microbiomes Distinguish Patients With Autoimmune Hepatitis From Healthy Individuals, Frontiers in Cellular and Infection Microbiology. (2020) 10, 10.3389/FCIMB.2020.00342/BIBTEX. PMC7416601 32850468

[54] Azam M. W. , Zarrilli R. , and Khan A. U. , Updates on the Virulence Factors Produced by Multidrug-Resistant Enterobacterales and Strategies to Control Their Infections, Microorganisms. (2023) 11, no. 8, 10.3390/microorganisms11081901. PMC10456890 37630461

[55] Kamboh A. A. , Shoaib M. , Abro S. H. , Khan M. A. , Malhi K. K. , and Yu S. , Antimicrobial Resistance in Enterobacteriaceae Isolated From Liver of Commercial Broilers and Backyard Chickens, Journal of Applied Poultry Research. (2018) 27, no. 4, 627–634, 10.3382/japr/pfy045.

[56] Janda J. M. and Abbott S. L. , The Changing Face of the Family Enterobacteriaceae (Order: Enterobacterales): New Members, Taxonomic Issues, Geographic Expansion, and New Diseases and Disease Syndromes, Clinical Microbiology Reviews. (2021) 34, no. 2, 1–45, 10.1128/cmr.00174-20. PMC8262773 33627443

[57] Lamba K. , Nelson J. A. , Kimura A. C. et al., Shiga Toxin 1–Producing Shigella sonnei Infections, California, United States, 2014-2015, Emerging Infectious Diseases. (2016) 22, no. 4, 679–686, 10.3201/EID2204.151825, 2-s2.0-84961203285. 26982255 PMC4806944

[58] O’Brien S. J. , The Public Health Impact of Food-Related Illness, Current Opinion in Infectious Diseases. (2012) 25, no. 5, 537–545, 10.1097/QCO.0B013E328356AEBA, 2-s2.0-84865865032. 22825290

[59] Troeger C. , Forouzanfar M. , Rao P. C. et al., Estimates of Global, Regional, and National Morbidity, Mortality, and Aetiologies of Diarrhoeal Diseases: A Systematic Analysis for the Global Burden of Disease Study 2015, Lancet Infectious Diseases. (2017) 17, no. 9, 909–948, 10.1016/S1473-3099(17)30276-1, 2-s2.0-85029667945. 28579426 PMC5589208

[60] Okoli C. , Anti-Microbial Resistance Profiles of *E. coli* Isolated From Free Range Chickens in Urban and Rural Environments of Imo State, Nigeria, Online Journal of Health and Allied Sciences. (2006) 5, no. 1, http://www.ojhas.org/issue17/2006-1-3.htm.

[61] Samanta I. , Joardar S. N. , Das P. K. et al., Prevalence and Antibiotic Resistance Profiles of Salmonella Serotypes Isolated From Backyard Poultry Flocks in West Bengal, India, Journal of Applied Poultry Research. (2014) 23, no. 3, 536–545, 10.3382/japr.2013-00929, 2-s2.0-84907023143.

[62] Ojo O. E. , Ogunyinka O. G. , Agbaje M. , Kehinde O. O. , and Oyekunle M. A. , Antibiogram of Enterobacteriaceae Isolated From Free-Range Chickens in Abeokuta, 2012, 14, Veterinarski arhiv, Nigeria.

[63] Cortés P. , Blanc V. , Mora A. et al., Isolation and Characterization of Potentially Pathogenic Antimicrobial-Resistant *Escherichia coli* Strains From Chicken and Pig Farms in Spain, Applied and Environmental Microbiology. (2010) 76, no. 9, 2799–2805, 10.1128/AEM.02421-09, 2-s2.0-77951551888. 20228098 PMC2863447

[64] Hasan B. , Sandegren L. , Melhus A. et al., Antimicrobial Drug–Resistant *Escherichia coli* in Wild Birds and Free-Range Poultry, Bangladesh, Emerging Infectious Diseases. (2012) 18, no. 12, 2055–2058, 10.3201/eid1812.120513, 2-s2.0-84869846439. 23171693 PMC3557866

[65] Lecoeur A. , Blanc F. , Gourichon D. et al., Host Genetics Drives Differences in Cecal Microbiota Composition and Immune Traits of Laying Hens Raised in the Same Environment, Poultry Science. (2024) 103, no. 5, 10.1016/j.psj.2024.103609. PMC11000118 38547541

